# Operative vs. conservative treatment of AC-Joint Dislocations Rockwood grade ≥ III -An economical and clinical evaluation-

**DOI:** 10.1186/s12962-023-00468-2

**Published:** 2023-09-13

**Authors:** Richard Niehaus, Alisa Schleicher, Elias Ammann, Philipp Kriechling, Christopher G. Lenz, Michael Masanneck, Sandro Hodel, Karim Eid

**Affiliations:** 1 Apollon Hochschule für Gesundheitswirtschaft, Apollon Hochschule für Gesundheitswirtschaft, Universitätsallee 18, 28359 Bremen, Germany; 2 Department of Orthopedic Surgery, Kantonsspital Baden, Im Ergel 1, Baden, 5404 Switzerland

**Keywords:** AC –Joint, Cost analysis, Rockwood, Outcome, Acromioclavicular Joint, I 15 Health and Economic development.

## Abstract

**Introduction:**

Acromioclavicular joint dislocations (ACD) are one of the most common shoulder injuries. There is no consensus in how to treat higher graded ACD ≥ Rockwood grade III. This study compares operative versus conservative treatment regarding costs and clinical outcome parameters.

**Materials and Methods:**

This retrospective, consecutive case-control-study includes 14 patients. Seven operatively treated patients were matched, by Rockwood grade, with seven conservatively treated patients. The cost was extracted out of the clinical- and insurance-based cost sheets and furthermore these include the loss of earnings. Clinical examination, demographic data as well as different outcome-questionnaires were recorded.

**Results:**

There were no significant differences between operative and conservative treated patients for outcome Questionnaires. Of note, there was a significantly higher incidence of tenderness over the AC-joint (p = 0.0038) postoperatively. As expected, economical evaluation showed various findings in favor of the conservative treatment. The costs for medical services (11012.39vs.1163.81USD; p = 0.0061), days of hospitalization (3.3vs.0days; p < 0.0001); total cost for medical treatment (30262.17 vs. 7833.82 USD; p = 0.0358) were significantly higher in the operative group.

**Conclusion:**

Even with a limited case number and a retrospective study design almost all clinical results were equal in both groups. Operative therapy of higher graded ACDs (Rockwood > III) compared to conservative is economically inefficient. Under consideration of clinical comparable results, indications for operative treatment should be set very carefully.

## Introduction

AC-joint dislocations are common. In literature, these injuries are counted among the most common shoulder injuries with a share of 9% [1–3]. In young individuals who play contact sports the significance of AC-joint dislocations is correspondingly greater, accounting for a large proportion of 30–50% of all shoulder injuries [2–5]. In 2013, Chillemi et al. showed that the incidence for AC-joint dislocation is 1.8 per 10 000 people per year in an urban population [1].

The most common used classification of AC-joint injuries today is the Rockwood classification. A total of six degrees of severity are distinguished [3, 5–8].

Despite the availability of many therapeutic options, the acute injury of the acromioclavicular joint and the choice of adequate therapy is still a great challenge today. There is a broad consensus regarding less severe injuries, i.e. type Rockwood grade I and II. Usually these are treated conservatively with excellent clinical outcome [5, 7] Treatment usually consists of a short-term phase of immobilization, followed by gradual mobilization. However, a uniform standardized procedure for conservative treatment does not exist and is the domain of the physiotherapist [3, 7].

Studies have shown higher grade injuries, type Rockwood grade IV-VI, which are associated with complete rupture of all ligament structures and therefore significant instability of the clavicle should be treated operatively [7, 9]. Data on conservative therapy attempts for Rockwood grade IV and V injuries are hardly available [10]. One factor in favor of operative treatment may be a remaining visible protrusion of the clavicle [11, 12]. There is a variety of operative treatments but there is no gold standard [14, 15]. The common techniques are either open surgery or arthroscopically assisted, for example using AC TightRope [11]. Open surgery includes fixation using the Bosworth screw [16], clavicle hook plate osteosynthesis [17] or the Weaver-Dunn autograft [18]. The fixation can be reinforced in each case by additive material (anchors, sutures). The method of choice depends mainly on the preoperative situation, the medical center and the surgeons experience [3, 13, 14].

The decision on how to proceed with Rockwood grade III injuries remains controversial. Recent studies recommend conservative therapy. Nevertheless, these injuries are up to day frequently operated. Decision making is here often influenced by patient-specific aspects (traumatic hematoma, initial pain, cosmetic aspects, sports-activities [3, 13, 14].

Due to the dilemma in choosing the optimal treatment options for higher-grade acromioclavicular joint dislocations, the aim of the study was to answer the following research question: Is it reasonable to perform surgical stabilization in a trauma level one public hospital of the acromioclavicular joint in cases of higher-grade acromioclavicular joint dislocation, considering economic and clinical points of view?

## Materials and methods

The data was collected at our level one trauma center, the study is a retrospective case-control study. All analysis conducted were provided after approval of the ethics committee in northwest- and central Switzerland (Swissethics ID 2020–02448). Patients included had AC-joint injuries Rockwood grade III - V between march 2017 and march 2021. Shoulder specialist performed all operations by athroscopic using the DogBone®-Button (Arthrex, Naples, FL, USA). In four patients, the acromioclavicular joint was additionally addressed by transfixation with two Swive-Lock® anchor (Arthrex, Naples, FL, USA) and a FiberTape® (Arthrex, Naples, FL, USA). Non-operative therapy consisted of immobilization in a sling or arm fixation vest for four weeks. Inclusion criteria were the age of > 18 years, Rockwood grade ≥ III and a follow-up of at least six months. Patients with further operations of the shoulder, degenerative or previous damages of the shoulder and rejection of participation were excluded.

### Costs

The total costs were calculated from the total medical services (including the surgery and hospitalization), medications, physiotherapy treatments, the nursing costs and the calculated loss of earnings. To obtain the data necessary for the cost report, the relevant service providers were requested in writing. From these, the data was meticulously included in the cumulative cost statement. Since this data does not reflect the cost of physiotherapy sessions performed, the average cost of a physiotherapy session for the respective group (operative / non-operative) was determined and multiplied by the number of physiotherapy sessions prescribed. The conversion of the Swiss Franc (CHF) into United States Dollar (USD) was done using the current monthly average rate of 1 USD to 0.9813 CHF in July 2022 [19].

### Clinical data

After identifying the participants and obtaining a written consent, we performed a clinical exam. Also we evaluated patient filled questionnaires. The Constant Score (CS) [20], which is a very frequent applied tool for assessment of the shoulder joint. It combines subjective and objective measurements such as pain, activities of daily living, strength and the range of motion. The Subjective Shoulder Value (SSV) defines patient’s subjective shoulder assessment, expressed as a percentage of a perfectly normal shoulder reaching 100%. It was also evaluated. The SF-36v2 (Short-Form 36, Version 2) is an outcome measure instrument to evaluate quality of life [21].Additionally the “Work Productivity and Activity Impairment Questionnaire: General Health” (WPAI:GH [22]) was used to evaluate the impairments in work and activities. Furthermore, data concerning radiographs including the determination of the Rockwood classification were collected. In addition, revision surgeries performed, the respective work incapacity, and hospitalization days were recorded. We determined from the clinical information system the cost unit, to determine the contact person for the cost reports as well as the number of performed consultation. Other factors included in the calculation of total costs were the number of X-rays taken, the number of physiotherapy sessions (including medical training therapy), the number of sick leave days and any medical aids purchased. The current clinical parameters were collected and recorded during the clinical examination, using a self-developed questionnaire. These were; range of motion (ROM) of both sides, strength of both sides, Pain using visual analog scale (VAS) and remaining tenderness over the AC-joint. In addition, questions were asked about subjective satisfaction with the outcome and the choice of therapy. As part of the clinical presentation, patients were asked the following questions about shoulder strain at work: “Do you perform shoulder-straining activities at work?“; “Do you carry heavy loads at work?“; “Do you experience repetitive shoulder strain at work?“; “How many months were you subjectively limited due to the shoulder? ».

### Statistical analysis

All data was collected in table form. The Kolmogorov-Smirnov test was used to assess any normal distribution. Normal distributed data, were represented by mean and standard deviation and analyzed using Student’s t-test. Abnormal distributed data, were analyzed considering median and range using the Mann-Withney-U test. Categorical data were compared using the Chi-square test and the Kruskal-Wallis test. The significance level was p < 0.05.

Statistical analysis of the data was performed using IBM SPSS® Statistics for Windows (version 22.0; IBM Corp., Armonk, NY, USA).

## Results

### Demographic and indication-specific results

Fourteen patients were included for the statistical analysis according to the before-mentioned criteria, seven patients received surgery and seven patients conservative treatment.

The 14 patients obtained were 13 men (93%) and one woman (7%). The mean age of both groups was not significantly different at the time of the study, although the patients in the surgery group were older in average (50.43 vs. 43.47 years, p = 0.341). In the surgery group, an average of 5.29 days (SD 2.98, range 1–11 days) elapsed between the time of the accident and the operation. The control group started therapy immediately after presentation to the emergency department, according to the schedule.

There was a significant difference between the two groups with regard to the period of follow-up. It was on average 2.55 times later in the surgery group (31 vs. 12 months, p = 0.004). This data is presented in Table 1.

**Table 1:** Patient demographics and radiological findings

.	Operative group (n = 7)	Non-operative group (n = 7)	P value
Male (n)	6	7	-
Female (n)	1	0	*-*
Age (years)	50.43 [15.80] (26, 70)	43.57 [9.25] (32,58)	0.3411
Time between accident and operation (days)	5.29 [2.98] (1, 11)	-	-
Time of follow up (months)	31.00 [13.60] (20, 50)	12.14 [4.38] (8, 21)	0.0044*
CC-distance before therapy (mm)	27.09 [4.31] (24, 36)	26.11 [3.31] (21.90, 30.20)	0.6418
CC-distance after therapy (mm)	13.09 [4.37] (7.70, 20.40)	23.64 [4.87] (14.60, 28.70)	0.0011*
CC-distance delta (mm)	14.00 [3.96] (8.70, 19.00)	2.47 [2.65] (-0.80, 7.30)	< 0.0001*
Revision surgery (n)	1	0	*-*

*CC*, coracoclavicular

Values are mean [standard deviation] (range)

* Significant p value for comparison of operative group vs. Non-operative group

(Chi-square test or Welch’s t-test)

Patients were “matched” according to the Rockwood classification, therefore the distribution of grades is identical: in the surgery group, there were three patients with Rockwood grade III, one patient with Rockwood grade IV, and three patients with Rockwood grade V.

The CC-distance in the radiographic examination at time of the last follow-up was on average significantly smaller in the surgical group than in the control group (13 vs. 24 mm, p = 0.001). No difference was detected in the average CC-distance between the two groups (27 vs. 26 mm, p = 0.64) before the start of the corresponding therapy.

**Fig. 1:**
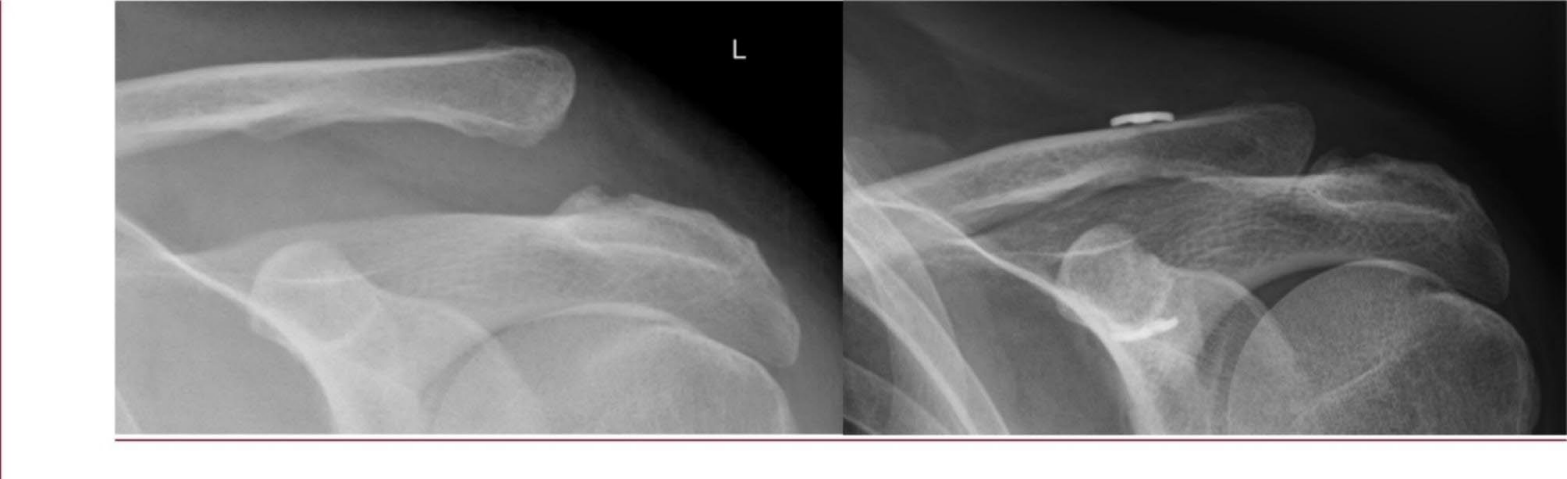
X-rays preoperatively/ like conservativly (left) and 6 Weeks postoperatively with high satisfaction

Clinical examination with Subjective Shoulder Value and Constant Score.

The clinical examinations show few differences with respect to both groups, Table 2.

**Table 2:** Results of the clinical examination of the acromiocavicular joint

	Operative group (n = 7)	Non-operative group (n = 7)	P value
Constant Score	89.14 [14.22] (60, 98)	88.57 [7.44] (73, 96)	0.9267
Constant Score sts.	98.57 [1.51] (96, 100)	97.14 [1.57] (96, 100)	0.108
Constant Score delta	-9.43 [13.95] (-38, 0)	-8.57 [7.79] (-25, 0)	0.8891
SSV (%)	89 [12.82] (65, 100)	75 [25.66] (20, 100)	0.2345
SSV sts. (%)	100 [0]	100 [0]	1
SSV delta (%)	-11 [12.82] (-35, 0)	-25 [25.66] (-80, -5)	0.2345
VAS	2.43 [3.26] (0, 8)	2.43 [1.90] (0, 5)	1
Pressure soreness over the AC-joint (total number «yes»)	5	2	0.0038*
Subjectively perceived satisfaction of therapy result (0–10)	7.14 [3.93] (1, 10)	8.43 [0.98] (7, 10)	0.4159
Force (newton)	140.21 [56.11] (76, 216)	139.90 [40.43] (82.50, 199.90)	0.9907
Force sts. (newton)	174.66 [48.57] (99.30, 236)	153.33 [30.78] (98.20, 193.80)	0.3458
Force delta (newton)	-34.44 [36.63] (-105, 1.80)	-13.43 [24.13] (-63, 8.90)	0.2291
Flexion (degrees)	162.88 [18.90] (120, 170)	170.00 [0]	0.3373
Flexion sts. (degrees)	170.00 [0]	170.00 [0]	1
Flexion delta (degrees)	-7.14 [18.90] (-50, 0)	0 [0]	0.3373
Abduction (degrees)	164.29 [15.12] (130, 170)	170.00 [0]	0.3374
Abduction sts. (degrees)	170.00 [0]	170.00 [0]	1
Abduction delta (degrees)	-5.71 [15.12] (-40, 0)	0 [0]	0.3374
External rotation (degrees)	60.71 [14.27] (45, 80)	59.29 [12.39] (45, 80)	0.8457
External rotation sts. (degrees)	65.71 [13.97] (50, 80)	60.71 [14.27] (45, 80)	0.5202
External rotation delta (degrees)	-5.00 [7.64] (-20, 0)	-1.43 [3.78] (-10, 0)	0.2895
Internal rotation delta (vertebrae)	-2.57 [2.51] (-5, 0)	-0.71 [1.89] (-5, 0)	0.1433

*AC*, acromioclavicular; *SSV*, Subjective Shoulder Value; *sts*., side-to-side, *VAS*: visual analog pain

Values are mean [standard deviation] (range)

* Significant p value for comparison of operative group vs. Non-operative group

(Chi-square test or Welch’s t-test)

In particular, a significantly more frequent tenderness over the AC-joint (p = 0.0038) was noted in the operated group. Five patients (71%) in the surgical group and two patients (29%) in the control group mentioned pain over the AC-joint during clinical examination. Perceived pain, objectified by VAS, was identical in both groups with a mean of 2.43 out of 10 points. The subjectively perceived satisfaction, with an average of 7.14 vs. 8.43 out of 10 possible points (p = 0.4159), was descriptively lower in the surgery group than in the control group, but not significantly. All patients in the control group would again prefer conservative therapy to surgical therapy. In the study group (surgery), six patients would be willing to undergo surgical therapy again. One patient, in the same preoperative situation, would not decide in favor of surgical therapy. Likewise, no significance was found regarding the Subjective Shoulder Value. However, the SSV of the affected shoulder tended to be higher in the surgical group (89% vs. 75%, p = 0.2345). Analogously, there was a tendency for the delta (difference) of the SSV in the side-to-side comparison (11% vs. 25%, p = 0.2345).

Concerning the Constant Score of the affected shoulder, the results were again comparable in both groups (p = 0.9267). With an average of 89.14 points (SD 14.2; range 60–98 points) in the surgical group and 88.57 points (SD 7.4; range 73–96 points) in the control group, no significance was found. The difference (delta) of the Constant Score in side-to-side comparison with the healthy shoulder, was slightly larger, i.e. in favor in the operated group than in the control group (9.43 vs. 8.57 points, p = 0.8891). However, these results were again not significant. Better range of motion were predominantly found in conservatively treated patients. Average flexion (163 vs. 170 degrees, p = 0.3373) and abduction (164 vs. 170 degrees, p = 0.3374) of the injured shoulder were slightly reduced in the surgery group compared to the control group. In lateral comparison (delta), external rotation (5.00 vs. 1.43 degrees, p = 0.2895) and internal rotation (2.57 vs. 0.71 vertebrae, p = 0.1433) were more limited in postoperative patients than in control group. The difference in force measured by measuring device in the side-to-side comparison (delta) was not significantly different, but shows the tendency of a stronger limitation in the surgical group (34.44 vs. 13.43 N, p = 0.2291).

### Cost analysis

For an overview, all the data collected for the cost analysis are presented in Table 3. In addition to the individual and shared costs determined, these tables also include the number of medical consultations; physiotherapeutic treatments; days of hospitalization; days of incapacity for work, in each case as the mean value from all patient data. There is a relatively large spread in the number of medical consultations of both groups, whereby the surgically treated patients were presented to the doctor on average almost two times more frequent (6.5 vs. 3.3 times), there is barely no statistical significance with a p value of 0.0798. One patient was in need of a revision surgery, which increased the total medical services for this patient accordingly.

We found a significantly higher level of service costs (p = 0.0061***)***, which is also reflected in the statistical significance of the evaluated parameter (p = 0.0061). Regarding the number of physiotherapeutic treatments determined, there were no significant differences between the mean cost (1 824.41 USD) of the surgical group and the conservatively treated patient group (1 034.80 USD) with p value of 0.0958.

The patients in the surgery group were incapacitated for work longer, in average for ten days (47.6 vs. 37.0 days, p = 0.4862). There was one pensioner per group. Two other patients did not require sick leave due to their injury. The daily income of both groups was not significantly different (p = 0.4862). The loss of earnings was calculated by multiplying the total number of sick leave by the calculated salary per day. The calculated loss of earnings of both groups is not statistically significant (p = 0.2264).

As shown in Table 3, the total costs of operated patients exceed the costs of conservative treated patients by a factor of more than 3.5 on average. This is reflected by the significantly different mean values (30 840.31 vs. 7 984.36 USD; p = 0.0358). It is also worth mentioning that none of the patients treated conservatively required hospitalization, whereas surgically treated patients were hospitalized for a mean of 3.3 days.

**Table 3:** Statistical comparison of relevant parameters operation vs. conservative group

	Operative group		Non-operative group		
		N		N	P value
Number of medical consultations	6.50 [4.14] (3;13)	7	3.29 [1.60] (2;6)	7	0.0798
Total medical services including surgery (USD)	11’012.49 [7’845.71] (5’026.51;26’460.76)	7	1’164.27 [228.84] (892.37;1’487.48)	7	0.0061*
Cost of medicines (USD)	103.26 [46.50] (72.41;156.74)	3	70.44 [41.17] (25.53;116.43)	6	0.2736
Number of physiotherapeutic treatments	37.50 [18.37] (18;72)	7	23.14 [10.21] (9;36)	7	0.0958
Cost of physiotherapeutic treatments (USD)	1’824.41 [935.26] (875.72;3’594.31)	7	1’034.80 [347.22] (410.90;1’345.21)	7	0.0582
Days of hospitalization (days)	3.33 [1.03] (2;5)	7	0 [0] (0;0)	7	< 0.0001*
Nursing cost (USD)	48.58 [27.00] (464.71;7’999.32)	7	37.71 [27.27] (4.28;67.67)	6	0.4862
Incapacity for work (days)	370.00 [363.32] (6;91)	6	205.65 [33.49] (0;75)	6	0.2564
Salary per day (USD)	19’138.70 [23’186.43] (84.92;1’019.10)	6	6’699.36 [4’610.02] (169.85;245.15)	6	0.2264
Calculated loss of earnings (USD)	30’262.30 [24’591.41] (509.55;59’107.80)	7	7’834.72 [5’026.33] (0.00;13’503.08)	7	0.0358*
Total cost (USD)	6.62 [4.22] (7’132.38;71’512.80)	7	3.35 [1.63] (1’582.05;15’929.14)	7	0.0798

*CHF*, Swiss Francs; *USD*, US Dollar

The conversion of CHF into USD was done using the current monthly average rate of 1 USD to 0.9813 CHF in July 2022

Values are mean [standard deviation] (range)

* Significant p value for comparison of operative group vs. Non-operative group

(Chi-square test or Welch’s t-test)

In summary, it is arguable that the patients in the study group tend to show economically inferior results in all areas. The mean values are in all areas more expensive of the surgical treated patients. Significances could be calculated in the areas “total medical services” (p = 0.0061), “hospital stay in days” (p < 0.0001) and “total costs” (p = 0.0358).

Outcome SF-36v2- and WPAI:GH-questionnaire.

The data obtained of the two groups were compared with each other and with the Swiss norm data and are presented in Table 4 [23].

**Table 4:** Tabular presentation of the results of the SF-36v2 questionnaire. Comparisons with each other, the operative group with the norm data of Switzerland and the non-operative group [23] [3]

	Operative group	Non-operative group	P value	Normdata operative group	P value	Normdata Non-operative group	P value
Physical health (%)							
	89 [16.69]	93 [12.54]	0.659	91.16 [17.01]	0.7718	91.16 [17.01]	0.7919
Limited physical							
conditional role function (%)	64 [47.56]	86 [37.80]	0.3693	86.41 [20.6]	0.0051*	86.41 [20.6]	0.929
Limited emotionally							
conditioned role function (%)	76 [41.79]	86 [37.80]	0.6628	87.64 [19.22]	0.1197	87.64 [19.22]	0.7926
Vitality (%)							
	70 [18.93]	71 [10.97]	0.933	63.24 [17.22]	0.3008	63.24 [17.22]	0.252
Mental health (%)							
	81 [21.00]	79 [17.39]	0.8286	75.02 [16.18]	0.3071	75.02 [16.18]	0.5315
Social functionality (%)							
	89 [28.35]	95 [9.83]	0.6456	85.84 [20.02]	0.6503	85.84 [20.02]	0.2455
Physical pain (%)							
	76 [25.93]	75 [29.63]	0.9439	74.52 [26.03]	0.8513	74.52 [26.03]	0.937
General health perception (%)							
	78 [17.99]	80 [13.84]	0.8072	75.64 [17.35]	0.7358	75.64 [17.35]	0.5071

Values are mean [standard deviation]

* Significant p value for comparison

(Chi-square test or Welch’s t-test)

No differences were detected in all categories of the SF-36v2 between the operation and the control group. In the categories “physical health”, “physical role function”, “vitality”, “mental health”, “social functioning”, “physical pain” as well as the general health perception are comparable in both groups with the norm data of Switzerland. Worth mentioning is only the significantly more limited emotionally conditioned role function (64 vs. 86.41%, p = 0.0051) of the surgery group, compared to the Swiss norm data.

The analysis of the WPAI:GH-questionnaire detected no significant differences in responses to the questions related to shoulder loading at work and activities between the surgical and control groups. Both groups were also comparable in terms of performing shoulder-loading activities (p = 0.2864), carrying heavy loads (p = 0.5000) and experiencing repetitive shoulder loads at work (p = 0.5000). Subjectively perceived limitations, although not significant on average, tended to be longer in the surgery group (13.71 vs. 4.43 months, p = 0.3636).

## Discussion

Economic burden on society for medical treatments is continuously increasing and therapeutic options should be more and more evaluated with respect to the potential costs in relation the clinical functional results [24, 25].

The main findings of this study are that both, the costs of medical services (11 012.39 vs. 1 163.81 USD p = 0.0061) and the total costs including loss of earnings (30 262.30 vs. 7 834.72 USD p = 0.0358) are significantly higher in the group of operated patients. These findings are in line with the results of Franovic et al. who reported a mean cost of 15 043 USD for the surgical and 6 077 USD for the conservative procedure [26]. The difference to the amounts of Franovic can be explained by the fact that only billing codes for clinical costs and mean income tables were used to estimate the income. Furthermore, physical therapy and other additional costs were not included in their study. In comparison, this study shows costs, which were largely abstracted from provider billing records and thus have a high degree of accuracy.

The costs of physiotherapy, as a main cost-factor, were abstracted from the billing documents of the service providers. If not possible, these were estimated in order to be included in the total cost accounting, this affected three patients in the surgical group and one in the operative group. It has to be mentioned here that the actual physiotherapy costs of these “estimated” patients are probably even higher, since only the prescriptions issued by our hospital were used for the calculation. Physiotherapy which were issued not in our clinic, for example by family physicians are not seized in the computation. However, the calculation (37.50 vs. 23.14 treatments) only included physiotherapies that could be determined by the billing receiptsand seems to be clearly higher. This is evidenced by the barely non-significant result in the comparison of both groups (p = 0.0582).

The hospitalization time was with the mean of 3.3 days significantly longer in comparison to the control group, which was completely out-patient treated.

The inclusion of a patient, needing revision surgery, in the cost analysis is to be discussed because it can be interpreted as an outlier. This can be justified with the extraordinarily high revision rate of AC-joint reconstructions as a whole. For example, Ochen et al. describes this as approximately 11% in a 2019 study [27]. This is roughly in line with the revision rate in this study of approximately 14%.

The main finding from the analysis of the clinical data was that surgical and conservative therapy of a higher-grade AC-joint injury Rockwood ≥ III were almost comparable in terms of clinical outcome. This was reflected with nearly identical mean Constant Scores of 89.1 points for the surgical group and 88.6 points for the control group (p = 0.9267). Longo et al. published a review in 2017 that included 14 other studies on the topic.

 [3, 28] Here, they also summarized comparable results for the Constant Score (87.3 vs. 88 points). A closer analysis of this study shows that only patients with AC-joint injury Rockwood grade III were considered. In another review by Chang et al. it was again postulated that there was no difference in functional outcome between surgical and conservative therapy for high-grade AC-joint injury [29]. In this study, patients from different therapy groups were not only compared but also additionally matched according to Rockwood grade. Despite the small patient population, this achieved good validity with regard to the thesis of an equivalent outcome between conservative and surgical therapy of a high-grade AC-joint injury [3].

The only significant difference was in the frequency of tenderness over the AC-joint in the clinical examination to the disadvantage of the surgical group, even though these patients had significantly later posttraumatic follow-up time (31 vs. 12 months, p = 0.0044). Therefore, an improvement in the further course seems unlikely. Regarding age, both groups are almost statistical comparable (50.43 vs. 43.47 years, p = 0.341). But a difference of nearly 7 years in average between both groups is noticeable. It seems that younger patients have more trust to their own physical body healing. However, outcome parameters are sustained comparable. Subjectively perceived pain was significantly more frequent in surgically treated patients (p = 0.004). Already in 2019, Koch showed that pressure soreness over the AC-joint was present postoperatively in 20% of patients. The study group presented an even significantly higher incidence of pressure soreness at 71.43% [30]. A 2015 review by Woodmass et al. showed that the most common postoperative complication, was irritation of the surrounding tissue by the implanted foreign bodies. About one-third of all patients treated arthroscopically with the TightRope or Endobutton technique were symptomatic [31]. Since all patients in the current study were treated arthroscopically using the DogBone-Button, this could be a reason for the more frequent occurrence of pressure soreness [3].

With regard to quality of life, no significant differences were found between the two groups in all categories of the SF-36-questionnaire. This was in line with the data in the literature, which is reflected in a review published in 2019. Here, quality of life was determined and compared with 357 patients [3, 32].

It should also be mentioned that the subjective satisfaction of patients in the control group with regard to the choice and outcome of treatment tended to be greater, but not significantly (7.14 vs. 8.43 out of 10 points, p = 0.4159). When asked if, given the same situation, the same choice of therapy would be made, one person in the surgery group declined to choose an operation again due to the lengthy follow-up. Interestingly, this was not the surgically revised patient, but one with an actually unremarkable postoperative course. Considering the statistical results of this study, it can be assumed that the patient-specific, subjective satisfaction of both groups was comparable, independent of the choice of therapy [3].

Finally, no significant differences were found between the surgical and control group with regard to the activity and work productivity of the patients, which was recorded by means of the WPAI:GH-questionnaire. Another measure to objectify work productivity was work disability, measured by the cumulative period of sick leave. This was not significant on average, but tended to be longer in the surgery group (48 vs. 37 days; p = 0.4862).

The subjective limitation in months perceived by the patients was also longer in the surgical group and additionally confirmed the objective finding (14 vs. 4 months, p = 0.3636). This might be partly due to the higher complication rate of surgical procedures, but another reason could be the fact that there be inhomogeneity in the severity of concomitant injuries despite Rockwood matching [29, 30] The review by Tamaoki et al. published in 2019 confirms that patients with conservative therapy appear to recover more quickly, as well as resume their work and activities sooner [32]. A small sample size as well as a high standard deviation possibly led to the fact that no significant difference was detectable in the present patient collective.

### Limitations

This study is subject to limitations. On the one hand, it is a retrospective study, the patients were therefore only included in the study after their treatment. Furthermore, the count of patients, 14 in number, is rather small which relativizes certain statements. Due to the clinical experience, which is also the basis of the research question of this thesis, the surgical indication has been set only cautiously on our part. This results in a small number of operated patients, which explains the manageable group size. Finally, the “matching” according to Rockwood classification should be mentioned. On the one hand, this is a clear advantage in comparison to study designs without matching, but additional matching taking into account gender and activity level would be desirable. However, this could not be realized in this study due to the small patient population.

## Data Availability

Data avalible on request due to restrictions e.g. privacy or ethical.
