# Correction to: Operative vs. conservative treatment of AC-Joint Dislocations Rockwood grade ≥ III -An economical and clinical evaluation-

**DOI:** 10.1186/s12962-023-00483-3

**Published:** 2023-10-09

**Authors:** Richard Niehaus, Alisa Schleicher, Ammann Elias, Philipp Kriechling, Christopher G. Lenz, Michael Masanneck, Sandro Hodel, Karim Eid

**Affiliations:** 1 Apollon Hochschule für Gesundheitswirtschaft, Universitätsallee 18, 28359 Bremen, Germany; 2 Department of Orthopedic Surgery, Kantonsspital Baden, Im Ergel 1, Baden, 5404 Switzerland

**Correction to: Cost Effectiveness and Resource Allocation (2023) 21:63**

10.1186/s12962-023-00468-2

Following publication of the original article [1], the authors flagged the following errors in the author list: the names of the fourth and fifth author had been erroneously mixed and combined into one name; the given name of the last author was shown as this author’s family name and vice versa. The author list has been corrected in the published article and the correct list may be seen in this erratum.

